# Susceptibility of Pets to SARS-CoV-2 Infection: Lessons from a Seroepidemiologic Survey of Cats and Dogs in Portugal

**DOI:** 10.3390/microorganisms10020345

**Published:** 2022-02-02

**Authors:** Ricardo Barroso, Alexandre Vieira-Pires, Agostinho Antunes, Isabel Fidalgo-Carvalho

**Affiliations:** 1 Department of Biology, Faculty of Sciences, University of Porto, Rua do Campo Alegre, s/n, 4169-007 Porto, Portugal; up201605888@edu.fc.up.pt; 2 Equigerminal, S.A., Rua Eduardo Correia, n°13 lote 20.12, 3030-507 Coimbra, Portugal; alexandre@equigerminal.pt (A.V.-P.); isabel@equigerminal.pt (I.F.-C.); 3 CNC-Center for Neuroscience and Cell Biology, CIBB, PhD Programme in Experimental Biology and Biomedicine (PDBEB) and Institute for Interdisciplinary Research, University of Coimbra (III UC), Rua Larga, 3004-504 Coimbra, Portugal; 4 CIIMAR/CIMAR, Interdisciplinary Centre of Marine and Environmental Research, University of Porto, Terminal de Cruzeiros do Porto de Leixões, Av. General Norton de Matos, s/n, 4450-208 Porto, Portugal; 5 CIVG - Vasco da Gama Research Center, Escola Universitária Vasco da Gama (EUVG), Campus Universitário, Av. José R. Sousa Fernandes, 3020-210 Coimbra, Portugal

**Keywords:** cats, coronavirus disease 2019 (COVID-19), diagnosis, dogs, epidemiology, one-health, SARS-CoV-2, zoonosis

## Abstract

Betacoronavirus (β-CoV) are positive single-stranded RNA viruses known to infect mammals. In 2019, a novel zoonotic β-CoV emerged, the severe acute respiratory syndrome (SARS)-CoV-2. Although the most frequent SARS-CoV-2 transmission route is within humans, spillover from humans to domestic and wild animals has been reported, including cats (*Felis catus*), dogs (*Canis lupus familiaris*), and minks (*Neovision vision*). In order to understand the potential role of domestic animals in SARS-CoV-2 global transmission, as well their susceptibility to infection, a seroepidemiologic survey of cats and dogs in Portugal was conducted. Antibodies against SARS-CoV-2 were detected in 15/69 (21.74%) cats and 7/148 (4.73%) dogs. Of the SARS-CoV-2 seropositive animals, 11/22 (50.00%) were possibly infected by human-to-animal transmission, and 5/15 (33.33%) cats were probably infected by cat-to-cat transmission. Moreover, one dog tested positive for SARS-CoV-2 RNA. Data suggest that cats and dogs are susceptible to SARS-CoV-2 infection in natural conditions. Hence, a one-health approach is crucial in the SARS-CoV-2 pandemic to understand the risk factors beyond infection in a human–animal environment interface.

## 1. Introduction

In December 2019, an outbreak of atypical pneumonia of unknown etiology was detected in the city of Wuhan, located at the Chinese province of Hubei [1]. The outbreak was caused by a novel betacoronavirus (β-CoV), severe acute respiratory syndrome (SARS)-CoV-2, responsible for the coronavirus disease 2019 (COVID-19) in humans [2]. The disease is assumed to be a viral zoonosis, as CoVs recovered from bats [3] and pangolins [4,5,6,7] show high genome identity with SARS-CoV-2.

As with all RNA viruses, SARS-CoV-2 is able to adapt to novel hosts via random genomic mutations subjected to natural selection, as mutants with higher host tropism within the quasispecies memory may be manifested during interspecies viral transmission [8]. Hence, although SARS-CoV-2 mainly circulates in the human population, surveillance of SARS-CoV-2 infection in animal species is crucial for a better understanding viral adaptation, evolution, and transmission on a wider scale.

Cats and dogs have been exposed to SARS-CoV-2, involving the detection of SARS-CoV-2 RNA in respiratory and/or fecal specimens [9,10,11,12,13], as well as specific SARS-CoV-2 antibodies [14,15,16,17,18,19,20,21,22,23,24,25,26,27,28]. According to experimental studies, dogs have little susceptibility to infection [29,30] in contrast with cats, who exhibited higher viral shedding, respiratory pathology, and efficient transmission of SARS-CoV-2 within cats by respiratory droplets [29,30,31,32]. Consequently, several questions arise about the role of pets in SARS-CoV-2 transmission and if they could become novel reservoirs of infection by reverse zoonosis due to prolonged contact with humans.

Here, we report a seroepidemiologic survey in Portugal from December 2020 to May 2021 on cats and dogs from a one health perspective that demonstrates the susceptibility of these pets to SARS-CoV-2 infection.

## 2. Materials and Methods

### 2.1. Sampling

All animals were sampled in 15 veterinarian clinicals, hospitals, and animal shelters across distinct regions of Portugal (northern, central, and south regions). Full signalment and clinical history from each animal were collected through epidemiological enquiries, including sex, age, weight, breed, geographic region, and clinical signs (asymptomatic, fever, respiratory, digestive, neurological, and others), as well as pet exposure to COVID-19. Pets in close contact with infected humans with a positive PCR result in a previous two-week COVID-19-positive household; pets in a COVID-19-negative household; pets in a COVID-19-suspected household; and pets’ exposure to outdoors. Informed consent was filled and signed by owners and/or veterinarians to collect animal data, blood samples, and swabs. Blood samples were collected in dry biochemical tubes or 2-mL microtubes without anticoagulant. Whenever possible, when seropositive or borderline results were observed, an additional sample was collected to address the duration of antibody responses in pets. For serum separation, the tubes were centrifugated at 500 rcf for 10 min and the supernatant was transferred to a clean 2-mL microtube. All swabs (oropharyngeal, nasal, and rectal) were collected by synthetic fiber swabs and immersed in 1.5 mL of viral transport medium. Sera and swabs were stored at −80 °C until processing.

### 2.2. Animal Data

**Figure A1:**
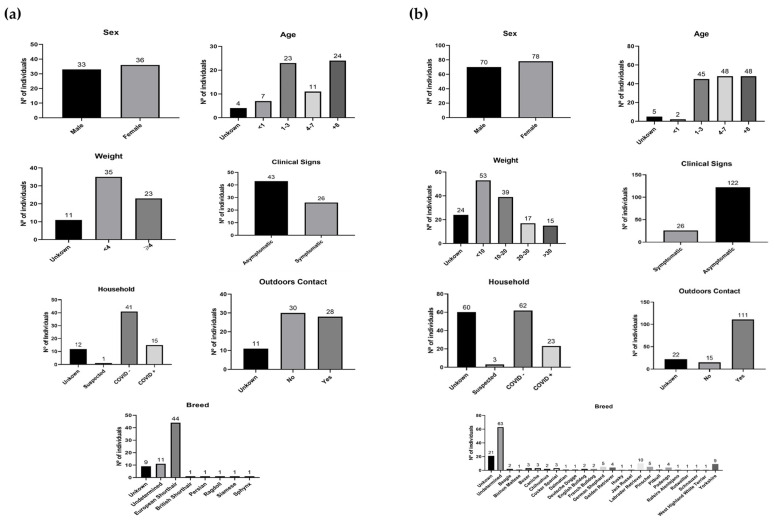
Signalment of (**a**) cats and (**b**) dogs from the SARS-CoV-2 seroepidemiologic study of pets in Portugal.

A total of 225 blood samples were collected from 217 pets (221 blood samples between 4 December 2020 and 8 May 2021 and four extra dog blood samples in June 2020), comprising a total of 148 dogs and 69 cats surveyed. Additional samples were collected from five dogs and three cats, totaling 72 cat and 153 dog blood samples. Oropharyngeal, nasal, and rectal swabs were collected from four dogs, with only nasal and rectal swabs collected from one cat. All cats (69/69) included in the survey lived in households. The surveyed dog population included 122 dogs from households and 26 from shelters (20 lived in close contact in parks, while 6 lived in individual cages). The data from pets signalment is present in supplementary information (Figure A1).

### 2.3. Survey Area

**Figure 1:**
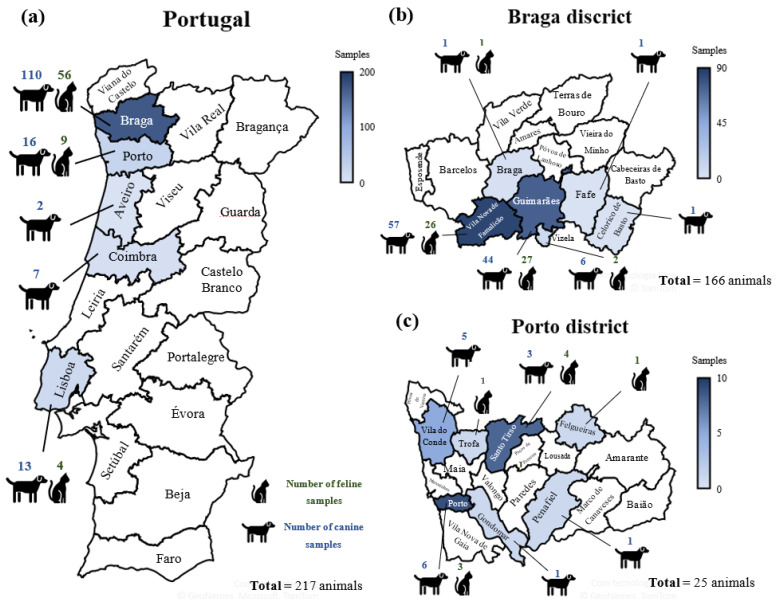
Geographic distribution of cat and dog samples, collected across (**a**) Portugal and in the regions of (**b**) Braga (Braga, Vila Nova de Famalicão, Guimarães, Vizela, Fafe, and Celorico de Basto as well as (**c**) Porto (Porto, Vila do Conde, Trofa, Santo Tirso, Gondomar, Felgueiras, and Penafiel).

The survey was performed in five districts of Portugal: Braga, Porto, Aveiro, Coimbra, and Lisbon. From the surveyed animals, 110 dogs and 56 cats were from the Braga district; 16 dogs and 9 cats were from the Porto district; 2 dogs were from the Aveiro district; 7 dogs were from the Coimbra district, and 13 dogs and 4 cats were from the Lisbon district. Most pet samples were collected at the Braga district (*n* = 166), mainly in the regions of Vila Nova de Famalicão (*n* = 83) and Guimarães (*n* = 71), followed by the Porto district (*n* = 26) (Figure 1).

### 2.4. Reverse Transcriptase Polymerase Chain Reaction and DNA sequencing

RNA extraction was performed using the Trizol LS reagent (Invitrogen) in 2-mL eppendorf DNA LoBind tubes according to the manufacturer instructions, with the portion of the RNA-dependent RNA polymerase (RdRp) and nucleocapsid (N) genes being amplified by RT-qPCR using the SARS-CoV-2 one step RT-qPCR kit (Nzytech) under the following cycling conditions: 50 °C for 20 min, 95 °C for 2 min, 40 cycles of 95 °C for 5 s, and 60 °C for 30 s. A small fragment of the spike (S) gene was also amplified by one step RT-qPCR using the following primers: S69d-F (ACA ACC ABA ACR CAA TTA CCC CC) and S69d-Rev (ACT CTG AAC TCA CTT TCC ATC CAA C) and sequenced by Sanger. Partial S sequences were analyzed by Clustal W by Dynamo software.

### 2.5. ELISA

Detection of IgG antibodies against SARS-CoV-2 RBD was accomplished by two ELISAs, each adapted for each species. The ELISA plate was coated with a recombinant RBD protein, diluted in phosphate-buffered saline with Tween^®^ detergent (PBST) and blocked using bovine serum albumin (BSA). All sera, including positive and negative controls, were screened at a dilution of 1:100 using a goat anti-cat or anti-dog IgG horseradish peroxidase (HRP) as a secondary antibody. The reactions ended with the addition of the HRP-substrate 3,3′,5,5′ Tetramethylbenzidine (TMB), followed by the 0.2 H_2_SO_4_ stop solution, resulting in switching from a blue color to yellow measurable signs. All the steps were separated by washes in a Wellwash^®^ Microplate Washer. The optical densities (ODs) were measured at 450 mm. The cutoff values were determined at three and five times the OD medium value of reactivity of seronegative samples from a pre-COVID-19 cohort. The test was considered valid if the quotient between the positive and negative control was above five.

### 2.6. Statistics

Data obtained from the epidemiologic surveys and results from the serologic analyses were registered in a Microsoft Excel spreadsheet, followed by the calculation of seroprevalences. Graph Pad Prism 8.0.1 was used for graphic construction and statistical analyses. Comparison between groups was performed using Fisher’s exact and Chi-squared tests. Significance was accepted when *p* < 0.05.

## 3. Results

### 3.1. SARS-CoV-2 Seroprevalence among Cats and Dogs

Antibodies against SARS-CoV-2 were detected in 22/217 (10.14%) pets, including 15/69 (21.74%) cats and 7/148 (4.73%) dogs. A total of 191/217 (88.02%) pets tested seronegative, and 4/217 (1.84%) had doubtful results. Booth species were prone to SARS-CoV-2 infection, with cats being more susceptible than dogs (Fisher’s exact test, *p* < 0.001). The detailed information about the seropositive pets is present in Table 1.

### 3.2. COVID-19 Households

Of the seropositive cats, 7/15 (46.67%) lived in COVID-19-positive households, 2/15 (13.33%) had contact with non-tested cats belonging to neighbors that were positive with COVID-19, 2/15 (13.33%) lived in the same COVID-19-negative household, and 1/15 (6.67%) lived with other non-tested cats in the same household. Of the seropositive dogs, 4/7 (57.14%) lived in COVID-19-positive households (Table 1). Cats were significantly more likely to test seropositive for SARS-CoV-2 if they had contact with COVID-19-positive human cases (Qui square test, *p* < 0.01), as well as dogs (Qui square test, *p* < 0.05) (Table 2).

### 3.3. Outdoors Access

From the seropositive pets, 7/15 (46.67%) cats and 6/7 (85.71%) dogs had contact with the outdoors (Table 1), but no significative differences were found regarding outdoor contact (Qui square test, *p* > 0.05) for both species in terms of infection (Table 2). Dogs belonging to animal shelters, 0/25 (0.00%), were seronegative to SARS-CoV-2.

### 3.4. Sex and Age

From seropositive cats, 6/15 (40.00%) were males and 9/15 (60.00%) were females, with 0/15 (0.00%) aged < 1, 6/15 (40.00%) aged 1–3, 5/15 (33.33%) aged 4–7, and 4/15 (26.67%) aged +8 years. From the seropositive dogs, 3/7 (40.00%) were males and 4/7 (60.00%) were females, with 0/0 (0.00%) aged < 1, 1/7 (14.29%) aged 1–3, 2/7 (28.57%) aged 4–7, and 3/7 (42.86%) aged +8 years (1/7 (14.29%) of unknown age) (Table 1). None of the surveyed kittens or puppies aged less than one year (*n* = 9) tested positive for SARS-CoV-2 antibodies, and no significative differences were found regarding sex (Fisher exact test, *p* > 0.05) or age (Qui square test, *p* > 0.05) in terms of infection (Table 2).

### 3.5. Clinical Signs

A total of 9/15 (60.00%) seropositive cats exhibited clinical signs, experiencing more than one of the following: 4/9 (44.44%) had respiratory signs, 2/9 (22.22%) had neurologic signs, 3/9 (33.33%) had digestive signs, 2/9 (22.22%) had apathy and appetite reduction, and 1/9 (11.11%) had fever. Additionally, 1/15 (6.67%) were co-infected with FIV. From these seropositive cats, 10/15 (66.67%) recovered from the clinical signs, while 5/15 (33.33%) were euthanized or succumbed to death. From the seropositive dogs, only 1/7 (14.29%) were symptomatic, exhibiting digestive signs (Table 1).

### 3.6. Viral Shedding, Seroconvesion, and Antibody Longevity

**Figure 2:**
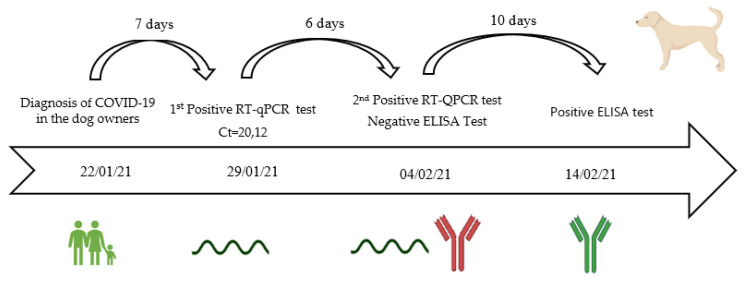
Timeline of the SARS-CoV-2 infection in an asymptomatic dog in a COVID-19-positive household. From the diagnosis of COVID-19 in the dog owners (22 January 2021) to the first and second positive RT-qPCR test of the dog on 29 January 2021 and 4 February 2021, respectively, and the positive ELISA test on 14 February 2021, 16 days after the first positive RT-qPCR test. The green color means positive serological or molecular result and the red color means a negative serological or molecular result. The date corresponds to the day of the blood or swab sampling from the dog.

In five animals, swabs for SARS-CoV-2 testing by RT-qPCR (four dogs and one cat) were available. Of these, only one asymptomatic dog tested positive for SARS-CoV-2 RNA seven days after the owner’s positive result was known, with a Ct value of 20.12 in a pool of three swabs (nasopharyngeal, oropharyngeal, and rectal swabs). The dog continued to shed the virus six days after the first swab, remaining positive for SARS-CoV-2 RNA with a Ct value of 31.57 for the oropharyngeal swab (positive), 28.71 for the nasal swab (positive), and >40 from the rectal swab (negative). The SARS-CoV-2 RNA positive dog was seronegative in the first blood sample, which coincided with the collection of positive swabs samples. However, after 16 days, antibodies against SARS-CoV-2 were already detected in a second blood sample (Figure 2).

In three of the seropositive animals, a second blood sampling was taken to investigate the longevity of anti-SARS-CoV-2 antibodies. One cat was seronegative for SARS-CoV-2 three months after testing seropositive, one dog was seronegative after one month, and one dog was seronegative after nine months.

### 3.7. Sequencing of a Short Fragment of SARS-CoV-2 S Gene

**Figure 3:**
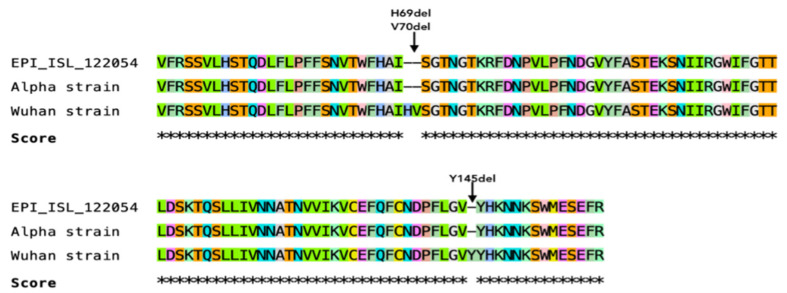
Clustal W analysis of partial Spike genome sequence retrieved in dog (EPI_ISL_122054) compared to Alpha and Wuhan strain.

A short fragment of the SARS-CoV-2 S gene was obtained by Sanger sequencing and deposited at the GISAID database under acession number EPI_ISL_1220542. The Clustal W analysis showed that the partial S sequence obtained from the dog was similar to the Alpha strain containing the H69del, V70del, and Y145del mutations (Figure 3).

## 4. Discussion

### 4.1. Animals Are Exposed to SARS-CoV-2

**Figure 4:**
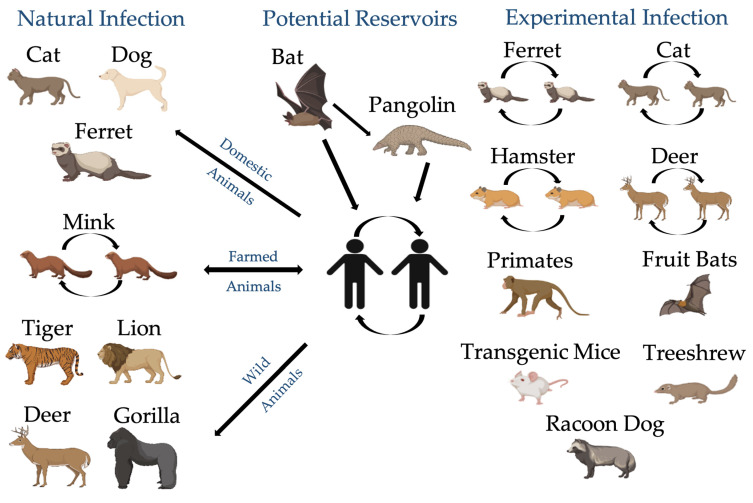
Possible transmission cycle of SARS-CoV-2 in different animal hosts. Created with BioRender.com.

Since the beginning of the COVID-19 pandemic, questions have remained about the susceptibility of animal species to SARS-CoV-2 infection under natural conditions and their potential role in viral transmission, as it is assumed as a viral zoonosis. Although diagnosis of SARS-CoV-2 is focused on humans, various molecular and serological assays were adapted to access SARS-CoV-2 infection in animals. Reports of SARS-CoV-2 infection were detected in domestic cats (*F. catus*) [10,11,12,13], dogs (*C. l. familiaris*) [9], and ferrets (*Mustela putorius furo*) [33], by closer contact with infected owners, as well in captivated animals, including minks (*N. vision*) [34], lions (*Panthera leo*), tigers (*P. tigris*) [33], and gorillas (*Gorilla gorilla)* [35], by closer contact with infected workers (Figure 4), confirming that viral spillover from humans to animals may be more common than expected. Viral spillover from animals to humans was only reported in mink farms [34], as mink-specific variants were developed due to rapid viral evolution and adaptation to animal hosts. Moreover, white-tailed deer (*Odocoileus virginianus)* [36] and minks (*N. vison*) [37] were found to be exposed to SARS-CoV-2 in the wild (Figure 4), creating potential new reservoirs of the virus.

Furthermore, experimental infections were reported that confirmed the susceptibility to SARS-CoV-2 infection and viral transmission in a wide range of animal species. Different species revealed varying levels of clinical outcomes and viral shedding, including domestic cats *(F. catus)* [29,30,31,32], dogs (*C. l. familiaris*) [29,30], ferrets (*M. p. furo*) [29,38,39,40], transgenic mice (*Mus musculus*) [41], Syrian hamsters (*Mesocricetus auratus*) [40], racoon dogs (*Nyctereutes procyonoides*) [42], Egyptian fruit bats (*Rousettus aegyptiacus*) [38], Chinese tree shrews (*Tupaia belangeris*) [43], white-tailed deer (*O. virginianus*) [44], rhesus macaques (*Macaca mulatta*) [45], and cynomolgus macaques (*M. fascicularis*) (Figure 3) [46], with some being resistant to infection, such as cattle (*Bos taurus*) [47], chickens, ducks, and pigs [29,38].

Previous reported data suggest that several animals are susceptible to SARS-CoV-2 infection in natural and experimental conditions.

### 4.2. Cats and Dogs Are Susceptible to SARS-CoV-2 Infection

Among the different animal species, cats and dogs may be more susceptible to SARS-CoV-2 for several reasons: (i) They are in close contact with humans; (ii) they are susceptible to other coronavirus infections, such as alphacoronavirus (α-CoV) (Feline CoV and Canine CoV) and β-CoV (Canine Respiratory CoV) infection [48,49]; and (iii) they are known to be susceptible to natural [9,10,11,12,13] and experimental [29,30,31,32] SARS-CoV-2 infection. Moreover, cats were reported to be not only susceptible to SARS-CoV-2 infection [50], but also capable of effectively transmitting SARS-CoV-2 through close contact between infected and naïve cats [29,30,31,32,50]. Henceforth, a one health approach to the current COVID-19 pandemic is important to better understand the risk factors at the human–animal environment interface. Thus, in the context of SARS-CoV-2 outbreaks, testing of dogs and cats in COVID-19 households could be important not only to ensure animal health and prevent viral evolution/adaptation within pets, but also to better assess the risk factors of the potential establishment of animal reservoirs and/or potential environmental contamination with SARS-CoV-2. 

In this wide-scale seroepidemiologic study, a total of 22/217 (10.14%) pets were seropositive for SARS-CoV-2, including 15/69 (21.74%) cats and 7/148 (4.73%) dogs, proving that both species were exposed to SARS-CoV-2 and are susceptible to infection. The seropositivity reported here was higher than the seropositivity rates recorded in humans in Portugal between May and June 2020 (2.9%) [51], demonstrating that pets are being infected with SARS-CoV-2 at considerable rates. However, the survey in humans was accomplished in the first months of the pandemic, where the incidence of infection was considerably lower than the incidence during the present study, which was conducted December 2020 to May 2021, coincident with the third COVID-19 wave [52].

### 4.3. Cats Are More Susceptible to SARS-CoV-2 Infection and May Transmit the Virus to Other Cats

Cats were significantly more susceptible to SARS-CoV-2 infection when compared to dogs (Fisher exact test, *p* < 0.001), confirming experimental studies that have highlighted cats as being more predisposed to infection [29,30,31,32]. Previous wide-scale studies supported the same findings, with cats displaying a higher SARS-CoV-2 seroprevalence. In Wuhan, China, 15/102 (14.70%) cats and 16/946 (1.69%) dogs were seropositive to SARS-CoV-2 [14,23]. In Italy, 11/191 (5.8%) cats and 15/451 (3.3%) dogs were seropositive to SARS-CoV-2 [25]. In Texas, USA, 41.2% of 17 cats and 11.9% of 59 dogs had detectable neutralizing antibodies against SARS-CoV-2 [27].

Furthermore, here we reported that cats living in COVID-19-negative households and with outdoor access were also found to be seropositive. Two cats from a COVID-19-negative household and one cat living with various non-tested cats from a COVID-19-negative household were seropositive for SARS-CoV-2 infection, as well as two cats who had contact with various non-tested cats belonging to neighbours who tested positive for SARS-CoV-2. The results suggest that cat-to-cat transmission is possible and may be more common than expected in natural settings, being consistent with experimental studies [29,30,31,32], where cat-to-cat transmission occurred.

### 4.4. Cats and Dogs from COVID-19 Positive Households Are at Higher Risk of SARS-CoV-2 Infection

No correlation was found between SARS-CoV-2 seropositivity and sex (Fisher exact test, *p* > 0.05), age (Qui Square test, *p* > 0.05), or contact with the outdoors (Qui Square test, *p* > 0.05) for both species. None of the seven kittens and two puppies (aged < 1 year) were seropositive for SARS-CoV-2, suggesting that adult cats and dogs are more prone to infection. Patterson et al. also demonstrated that none of the 9 kittens and 20 puppies tested were infected by SARS-CoV-2 [25], revealing that younger pets may be less likely to be infected. 

However, both cats (Qui square test, *p* < 0.01) and dogs (Qui square test, *p* < 0.05) were more susceptible to SARS-CoV-2 infection if they had contact with COVID-19-positive human cases. In surveys conducted in Wuhan, the highest neutralization titers were observed in cats and dogs from COVID-19-positive households [14,23], revealing that pets are at a higher risk when in close proximity to infected owners. In addition, Fritz et al. also reported a higher seroprevalence in animals from COVID-19-positive households (21% to 53%, depending on the positivity criteria chosen), and infection was not associated with the number of pets in the households [28], suggesting that the main source of SARS-CoV-2 infection resides in pet owners. 

In this study, one dog was shown to be infected with SARS-CoV-2 seven days after contact with COVID-19-positive owners. Furthermore, in this asymptomatic dog, viral shedding was detected in a second RT-qPCR (with low Ct values), six days after the dog’s first positive test and 13 days after the owner’s positive COVID-19 test. The data suggests that SARS-CoV-2 is able to replicate in asymptomatic dogs for almost a week.

### 4.5. The Environment as a Potential Source of SARS-CoV-2 Infection of Cats and Dogs

All dogs belonging to animal shelters, 0/25 (0.00%), were seronegative to SARS-CoV-2 infection, with 20 living in close contact in parks and six kept in separated cages. Hence, it is unlikely that dogs from shelters are more prone to SARS-CoV-2 infection, as this infection was more prevalent within COVID-19-positive households. However, a SARS-CoV-2 seropositivity of 2.1% was reported in Dutch shelter cats [17], evidencing that cats may be more susceptible to SARS-CoV-2 infection in these conditions due to efficient viral transmission between cats. Moreover, reports of stray cats and dogs seropositive for SARS-CoV-2 were also described, evidencing the role of cat-to-cat transmission and/or environmental contamination as a potential source of SARS-CoV-2 infection [15,16,22].

In this study, 4/15 (26.67%) of the seropositive cats and 2/7 (28.57%) of the seropositive dogs lived in negative households but had outdoor access, substantiating the potential exposure to contaminated environments or to other infected animals.

### 4.6. Viral Coinfection can Lead to a Higher Susceptibility of Animals to SARS-CoV-2 Infection

Of the seropositive cats, 1/15 (6.67%) were co-infected with FIV and was symptomatic. Villanueva-Saz et al. reported that 3/4 (75.00%) cats seropositive for SARS-CoV-2 were co-infected with *Toxoplasma gondii*, FIV, or both [16]. It is therefore hypothesized that coinfection with FIV may play an important role in the susceptibility and clinical outcome of SARS-CoV-2 infection in cats.

### 4.7. Clinical Signs of SARS-CoV-2 Seropositive Cats and Dogs Are Innespecific and Variable

A higher percentage of seropositive cats were symptomatic, including 9/15 (60.00%) cats, with most showing more than one clinical sign, and 5/15 (33.33%) were euthanized or succumbed to death. On the contrary, from the 7 seropositive dogs, only 1 (14.29%) was symptomatic and exhibited digestive signs. Hence, cats appear to be more likely to develop disease and death associated with SARS-CoV-2 infection than dogs. However, more studies are needed to confirm this finding.

Previous reports showed that cats and dogs infected with SARS-CoV-2 suffered from myocarditis [53] and three cats developed respiratory signs, loss of appetite, and lethargy [11,13]. However, a longitudinal study in Texas of dogs and cats living with at least one human infected with SARS-CoV-2 reported that the majority (82.4%) of SARS-CoV-2-infected cats and dogs were asymptomatic [27], demonstrating different results.

### 4.8. Humoral Immunity of Cats and Dogs to SARS-CoV-2 Infection May Not Be Very Lasting

Evaluation of seroconversion and antibody longevity in pets is crucial to evaluate humoral responses to SARS-CoV-2 in natural conditions. The RT-qPCR positive dog in this study was seropositive 16 days after the first positive RT-qPCR result of the dog, however the time to seroconversion is unknown. In experimental studies, dogs seroconverted 14 days post infection (dpi), with a peak at 21 dpi [30], and cats at 7 to 12 dpi [29,30]. Pets seroconversion data are similar to the results observed in humans, where individuals seroconverted at 13 to 21 dpi [54]. Therefore, for diagnostic purposes, serological testing of pets should be performed at least two weeks after contact with a COVID-19-positive owner.

Moreover, in this study, the results of a second sampling of one cat and two dogs suggest that longevity of antibodies against SARS-CoV-2 may not be lasting, as one cat was seronegative after three months and two dogs were seronegative after one and nine months. Remarkably, consistent persistence of SARS-CoV-2 specific antibodies was reported in one pet ferret 129 days after the first sampling [55], however extensive longitudinal animal studies are lacking to address antibody longevity. Hamer et al. reported that the titers of neutralizing antibodies in pets seem to fluctuate along time, as across 15 antibody-positive animals, titers increased (33.3%), decreased (33.3%), or were stable (33.3%) over time [27]. Therefore, more studies are needed to better understand humoral responses to SARS-CoV-2 by pets.

### 4.9. Viral Evolution May Be Associated with SARS-CoV-2 Adaptation into New Hosts

While no study to date revealed transmission of SARS-CoV-2 from pets back to humans, cats and dogs demonstrated susceptibility to SARS-CoV-2 infection and may therefore represent potential viral reservoirs. As the S protein of SARS-CoV-2 is under strong positive selection [56]. A number of mutations in the S protein not found in viruses recovered from humans were found in viruses recovered from animals [57], evidencing that these can be associated with increased adaptability/variability to other animal hosts and give rise to new animal-specific variants, as reported in mink [34]. Therefore, a one-health approach to the pandemic is crucial to prevent the emergence of new animal-specific variants as well as to better understand viral-host adaptation and evolution processes of SARS-CoV-2.

## 5. Conclusions

The importance of animals in the transmission of zoonotic diseases is often difficult to determine, as data on these zoonotic viruses in animals are generally poor. The role of animals in important zoonoses, such as SARS-CoV-2 can be elucidated through large-scale seroepidemiologic studies using a one-health approach to identify events at high-risk interfaces, such as a human–pet interface, which may contribute to the spread and perpetuation of these diseases. In this study, we demonstrated that cats and dogs were susceptible to SARS-CoV-2 infection primarily through close contact with infected owners, with cats appearing more susceptible to infection than dogs. The data obtained emphasize the need for further epidemiologic studies, and the need to implement animal surveillance plans for SARS-Co-2 in animals in the future. To the best of our knowledge, this is the first report of a wide-scale seroepidemiologic study of SARS-CoV-2 infection in cats and dogs under natural conditions in Portugal.

## Figures and Tables

**Table 1:** Data from the SARS-CoV-2 seropositive pets.

Spp.	Date of Sample Collection	Sex	Age	Breed	Weight (kg)	COVID-19 Household Status	Outdoors Contact	Clinical Signs and Other Viral Infections	Date of Reported Clinical Signs	Recovery	Location
**Cat**	15 Dec.20	M	3	ES	5.0	Negative *	Yes	A; RA	01 Jan.21	No (Death)	Guimarães
**Cat**	15 Dec.20	F	10	ES	3.0	Positive	No	A; RA; Pyometra; FIV	01 Jan.20	No (Death)	Vizela
**Cat**	15 Mar.21	M	4	ES	4.6	Positive	Yes	No	NA	Yes	VNF
**Cat**	18 Mar.21	F	4	ES	3.0	Positive	No	R	18 Mar.21	Yes	VNF
**Cat**	17 Mar.21	F	10	Ragdoll	5.3	Negative *	Yes	N; R	17 Jan.21	Yes	VNF
**Cat**	26 Feb.21	F	2	ES	4.5	Negative	Yes	D; Seborrhea; Ulcers	18 Feb.21	Yes	Guimarães
**Cat**	26 Feb.21	F	2	ES	3.8	Negative **	Yes	D	25 Feb.21	No (E)	Guimarães
**Cat**	11 Mar.21	F	5	ES	5.0	Negative	Yes	D; F; R	05 Mar.21	Yes	VNF
**Cat**	15 Mar.21	F	16	Und	4.0	Negative ***	No	No	NA	No (Death)	Guimarães
**Cat**	19 Mar.21	F	2	ES	3.4	Positive	No	No	NA	Yes	VNF
**Cat**	18 Mar.21	F	1	ES	3.3	Positive	No	No	NA	Yes	Braga
**Cat**	27 Mar.21	M	4	Und	2.9	Negative ***	No	N	27 Mar.21	No (Death)	Guimarães
**Cat**	29 Mar.21	M	2	Und	3.5	Negative	Yes	No	NA	Yes	Guimarães
**Cat**	08 Apr.21	M	7	Persian	3.7	Positive	No	No	NA	Yes	Guimarães
**Cat**	19 Apr.21	M	15	NA	NA	Positive	NA	R	NA	Yes	Lisbon
**Dog**	05 Jun.20	M	8	NA	NA	Negative	Yes	No	NA	NA	Lisbon
**Dog**	14 Mar.21	F	10	LR	34	Negative	Yes	D	14 Mar.21	NA	Guimarães
**Dog**	26 Mar.21	F	12	Und	9.5	Positive	Yes	No	NA	Yes	Guimarães
**Dog**	26 Mar.21	M	7	Und	7.0	Positive	Yes	No	NA	Yes	Guimarães
**Dog**	04 Feb.21	F	NA	NA	NA	Positive	Yes	No	NA	NA	Coimbra
Dog ****	14 Feb.21	F	4	NA	NA	Positive	Yes	No	NA	NA	Coimbra
**Dog**	09 Feb.21	M	1	NA	NA	NA	NA	No	NA	NA	Lisbon

Abbreviations: * Contact with cats belonging to neighbors’ positive for COVID-19; ** Contact with other cats (not tested) in the same household; *** Cats belonging to the same household; **** Dog positive for SARS-CoV-2 RNA; A- Apathy; D- Digestive signs; E–Euthanized; ES–European Shorthair; F- Fever; FIV–Feline immunodeficiency virus; LR–Labrador Retriever; N–Neurologic signs; NA-No available information; R- Respiratory signs; RA–Reduced appetite; Und–Undetermined; VNF–Vila Nova de Famalicão.

**Table 2:** Risk factors contributing to cats and dogs SARS-CoV-2 seropositivity.

Risk Factor	Cats			Dogs		
	N°+ (Total)	%	*p*	N°+ (Total)	%	*p*
**Household**			0.004			0.020
COVID19**^+^**	7 (15)	46.67%		4 (23)	17.39%	
COVID19**^−^**	6 (41)	14.63%		3 (62)	4.83%	
Suspected	0 (1)	0.00%		0 (3)	0.00%	
Unknown	0 (12)	0.00%		1 (60)	1.67%	
**Outdoors**			0.448			0.586
Yes	7 (30)	23.33%		6 (108)	5.56 %	
No	7 (28)	25.00%		0 (15)	0.00 %	
Unknown	1 (11)	9.09%		1 (25)	4.00 %	
**Sex**			0.567			>0.999
Male	6 (33)	18.18%		3 (70)	42.86%	
Female	9 (36)	25.00%		4 (78)	51.28%	
**Age**			0.816			0.125
<1	0 (7)	0.00%		0 (2)	0.00%	
1–3	6 (23)	26.09%		1 (45)	2.22%	
4–7	5 (11)	36.36%		2 (48)	4.17%	
8+	4 (24)	16.67%		3 (48)	6.25%	
Unknown	0 (4)	0.00%		1 (5)	20.00%	

## Data Availability

The data presented in this study are available on request from the corresponding author.
